# Feeding practices of caregivers with children attending early childhood development centres in Xhariep, South Africa

**DOI:** 10.4102/hsag.v29i0.2575

**Published:** 2024-07-02

**Authors:** Angelique C. Carson-Porter, Violet L. van den Berg, Ntsoaki L. Meko

**Affiliations:** 1 Department of Nutrition and Dietetics, Faculty of Health Sciences, University of the Free State, Bloemfontein, South Africa

**Keywords:** child health, mothers, poverty, rural, unemployment, educational level

## Abstract

**Background:**

In low- to middle-income countries, malnutrition is a major contributing factor in children failing to achieve their developmental potential. The prevention of malnutrition requires, among others, nutritious, diverse and safe foods in early childhood.

**Aim:**

The study aimed to determine primary caregivers’ choices and motivation for the foods they fed their children.

**Setting:**

The study was conducted among early childhood development centres in the Xhariep District, Free State.

**Methods:**

A qualitative study was undertaken. Twelve participants who met the inclusion criteria were conveniently sampled. Semi-structured interviews were conducted to find out the primary caregivers’ choices and motivation for foods they fed their children until data saturation was reached.

**Results:**

The mean age of the participants was 31 years. Nine of the participants relied on social grants as a source of income. The participants reported feeding their children mainly maize porridge, milk, juice, and water. Vegetables and meat were fed to the children once a week. Fruits were fed to the children at the beginning of the month.

**Conclusion:**

The level of education, employment status, and community support influenced the primary caregivers’ feeding practices. The content of the diets of their children was insufficient in vegetables and fruit, not only placing the children at risk of undernutrition but also at risk of obesity and micronutrient deficiencies. Primary caregivers ensured their children were fed, although limited foods were offered.

**Contribution:**

This research creates awareness of the level of social progress and access to resources within rural communities in the Xhariep district, and gives the opportunity to extend this research to confirm these findings in other poverty-stricken areas.

## Introduction

Malnutrition in young children remains a global challenge that is not appropriately prioritised (Madiba, Chelule & Mokgatle 2019). In 2008, at least 200 million children in low- to middle-income countries failed to achieve their developmental potential because of malnutrition as a major contributing factor (Mayneris-Perxachs & Swann 2018). Malnutrition was responsible for half of all childhood deaths globally, and it was a leading contributing factor to illnesses such as pneumonia, diarrhoea, and malaria, and is a significant factor in slowed childhood cognitive development (UNICEF 2009:12). In 2019, according to the State of the World’s Children Report, 149 million of the children below the age of 5 years were stunted, 49.5 million were wasted, and at least 340 million children (one in two) suffered from hidden hunger (UNICEF 2019:12). Hidden hunger refers to ‘micronutrient deficiencies caused by insufficient intake of vitamins and minerals (including vitamin A, iron, zinc and iodine), essential for growth and development’ (May et al. 2020:23). In the Southern and Eastern African Region, 29 million children were stunted, and 5.4 million were wasted. Addressing childhood malnutrition in South Africa remains a huge challenge (May et al. 2020:25). Therefore, policies and programmes that promote appropriate dietary intake and lifestyles to prevent and manage malnutrition are critical initiatives to improve food and nutritional security (Schönfeldt, Hall & Bester 2013:233).

The nutritional status and health of children younger than 5 years of age are considered indicators, which reflect social progress, development, and access to resources within communities (Shisana et al. 2013:39). In developing countries, undernutrition is a major contributing factor to children failing to achieve their developmental potential (Mayneris-Perxachs & Swann 2018:1). The prevention of malnutrition requires, among others, safe, nutritious, and diverse foods in early childhood (UNICEF, WHO & World Bank 2019). The 2016 South African Demographic and Health Survey reported that 27% of children younger than 5 years were stunted. Young children become vulnerable to malnutrition when complementary foods are introduced and breastfeeding is discontinued (Sayed & Schönfeldt 2020).

In the long term, early malnutrition, particularly in the ‘First 1000 Days’ leads to poor child development and impaired learning capacity (Black et al. 2013; Mayneris-Perxachs & Swann 2018). In addition, the physiological impact of malnutrition remains into adolescence and adulthood, increasing susceptibility to central obesity, insulin resistance, and the associated metabolic changes that may eventually culminate in non-communicable diseases (NCDs) like type 2 diabetes, hypertension, and dyslipidaemia (Soliman et al. 2021:6). The risk of overweight, obesity and NCDs is worsened by the greater availability of low-cost energy from sugary and fatty foods. Adults who were stunted at a young age are also more likely to have lower work capacity and lower incomes (Jenkins 2015:3; Steenkamp, Lategan & Raubenheimer 2016:27). Moreover, malnutrition has an intergenerational effect as malnourished mothers often have malnourished children (May et al. 2020:30).

Undernourished children experience food insecurity at some point in their lives, particularly in situations of poverty, single-parent homes, child-headed families, and unemployment (Schwarzenberg & Georgieff 2018:2). These factors threaten food accessibility and food utilisation. The South African National Health and Nutrition Examination (SANHANES) reported that 45.6% of the South African population were food secure, 28.3% were at risk of hunger, and 26.0% were food insecure. Most food insecure participants lived in rural (37.0%) and peri-urban (32.4%) areas. The highest prevalence of food insecurity occurred among the black African population (30.3%), followed by the mixed race population (13.1%). Children often skip meals or do not eat food all day because of food insecurity (Schwarzenberg & Georgieff 2018:3). Moreover, compared to male-headed households, households with young children that are female-headed (25%) are more likely to reduce meal sizes because of food insecurity (17.3%) (STATS-SA 2016:13). According to the General Household Survey conducted in South Africa during 2016, 7.2 million children aged 0–6 years lived in households in which meals were skipped because of insufficient food in the house (STATS-SA 2016:38). Moreover, the limited types of food that poor households can afford limit dietary diversity, which is a major driver of hidden hunger (Schönfeldt et al. 2013:233).

The prevention of all forms of malnutrition requires:

[*A*]dequate maternal nutrition before and during pregnancy and lactation, optimal breastfeeding in the first two years of life, nutritious, diverse and safe foods in early childhood, and a healthy environment, including access to basic health, water, hygiene and sanitation services, as well as opportunities for safe physical activity. (UNICEF et al., 2019:2)

A healthy diet is essential for growth and development and is crucial for long-term and short-term disease prevention (Hamilton, McIlveen & Strugnell 2003:113). Because access to food and food utilisation differ between populations (Contento 2011:32–33), a healthy diet is threatened by poverty, the leading cause of food insecurity among millions of children (Schönfeldt et al. 2013:233; Steenkamp et al. 2016:27) particularly in children under the age of 5 (May et al. 2020:25).

Only the (quantitative) *Assuring Health For All in the Free State* (AHA-FS) study, under the auspices of the University of the Free State, has focussed on nutritional issues in the Kopanong area. This study found that among a convenience sample of 40 infants and preschool children, 47.5% were stunted, 15.0% were underweight, and 6.0% were wasted (Tydeman-Edwards et al. 2018:8). Thus, this rural area has already confirmed malnutrition among young children. The Faculty of Health Sciences of the University of the Free State has been doing community-based education and interprofessional training by rendering health services to the communities of the Xhariep district. During this time, the university’s Department of Nutrition and Dietetics noticed that mothers of young children face many barriers to feeding these children an appropriate diet.

Moreover, this study answers the call of Mokone, Manafe and Ncube (2023) to further necessary studies conducted at early childhood development (ECD) centres in other South African provinces among mothers and primary caregivers to assess their feeding practices. Therefore, this qualitative study aimed to explore the feeding practices of mothers with children between the ages of 2 and 6 years who attend ECD centres in South Africa in Jagersfontein, Springfontein, and Trompsburg. The objectives of this study were to explore which foods the mothers fed their children and whether they considered these foods nutritious.

This study stems from a published thesis submitted in partial fulfilment of ‘Magister Scientia in Dietetics in the Faculty of Health Sciences, Department of Nutrition and Dietetics, University of the Free State, Bloemfontein’.

## Methods

### Study design

An exploratory qualitative study using a phenomenology approach focussing on the experiences and perspectives of the mothers was needed to understand the factors that affect the feeding practices of mothers with young children in the area. The data were collected using semi-structured, face-to-face interviews in the rural towns of the Xhariep District, Free State province, South Africa, in March 2021.

### Study setting

The Xhariep district in the southern part of the Free State province is a rural province with a smaller population than most others in South Africa (Statistics South Africa [STATS- SA] 2021).

### Study population

The study population consisted of mothers and primary caregivers aged 18 years and above, of children between the ages of 2 and 6 years attending ECD centres in March 2021 from the three rural towns of Trompsburg, Jagersfontein, and Springfontein. These are rural towns in South Africa affected by unemployment. Selection criteria encompassed residency within these areas. According to the Department of Social Development in the Xhariep district, there are five ECD centres in Jagersfontein, two in Springfontein, and four in Trompsburg. All were visited by the researcher to secure permission to conduct the study at their ECD centres from the matrons, with all participants providing written informed consent.

### Sampling and recruitment

Convenience sampling, aligning with phenomenology research seeking fundamental truths, was used as the population selected was easily and conveniently available (Maree & Pietersen 2016:197). The mothers and primary caregivers were the unit of analysis for this study (Babbie 2016:9). The principals (also referred to as heads or directors) of the ECD centres were referred to as matrons, were asked to notify eligible mothers and primary caregivers about the study during researcher visits. Twelve participants, three from Trompsburg, three from Springfontein, and six from Jagersfontein, volunteered for interviews at the respective ECD centres their children attended in March 2021.

### Study instrument

A self-developed interview schedule based on literature was used to obtain data from the mothers about their lived experiences in feeding their children. The questions that were asked during the interview aligned with the study’s objectives. Semi-structured interviews were conducted with the participants, who described their lives, culture, beliefs, norms, knowledge, practices, and living environment related to feeding, food procurement, and preparation practices they applied to their young children and how they influenced their feeding practices; shedding light on the influences shaping their feeding habits. This method facilitated a comprehensive exploration and understanding of the participants’ perspectives on the significance of the meaning of food and which foods were considered nutritious.

### Procedures for data collection

As the data collection occurred in March 2021, during the coronavirus disease 2019 (COVID-19) pandemic, the appropriate COVID-19 protocols were observed.

### Data analysis

The researcher transcribed interviews and used NVivo 12 Pro software for analysis (NVivo 2020:1), employing data reduction techniques such as content analysis (Margolis & Zunjarwad 2018:620), to frame participants’ feeding practices. Open and descriptive coding led to themes and sub-themes merged from codes (Schurink, Fouché & De Vos 2018:405–406). Analysis continued until no new major themes emerged (Maree 2016:42–43; Perkins, Cunningham & Taveras 2015). Both interview schedules and observations and reflections were independently coded in NVivo. The data were summarised and reported with direct quotes to illustrate the findings. This research process involved emerging questions and procedures, on-site data collection, and interpretation from specific to broader themes. Interview guides were initially in English and were later translated into the local languages of Afrikaans and Sesotho for clarity.

### Trustworthiness

The researcher collected data through audio-recorded interviews, transcribing them verbatim immediately after each interview on the same day for enhanced trustworthiness. Verbatim transcripts were translated into English for analysis and meticulously reviewed line by line to grasp context and meanings, fostering deeper engagement with the data. Credibility was ensured by using participants’ quotes contextually to support arguments. Addressing study limitations provided clarity on the findings and conclusions drawn. The focus on understanding participant perspectives ensured transferability, emphasised by detailed descriptions of the population and procedures for data collection, facilitating application to similar rural populations. To ensure dependability, data evaluation and interpretation involved ongoing consultations with supervisors. As per Denzin and Lincoln (2000), informal participant consultations further validated data accuracy, ensuring confirmability.

### Ethical considerations

This study was approved by the Health Sciences Research Ethics Committee, Faculty of Health Sciences, University of the Free State (UFS-HSD2020/1821/2302), and the Free State Department of Social Development. Furthermore, all the participants gave written informed consent before participation in the study. Data were deidentified to protect confidentiality.

## Results

Demographic characteristics of the participants are described in Table 1. The primary caregivers who participated in this study cared for children aged 2–6 years. The youngest participant was 20-years old, and the oldest 71-years old. The median (25th–75th) age was 31 (26.8–41.8) years. Of the 12 participants, 8 had only one child in their primary care, while the rest were the primary caregiver of 2–3 children. Only 5 participants (*n* = 12) had Grade 11 or 12 school education, while 6 had different levels of primary school education, and 1 never attended school. Most of the participants were not married, but in a relationship. Eight of the participants received a child support grant, while two received pension grants. Only two participants had a spouse or partner who was employed and one participant was employed. More than half of the participants resided in nuclear families, consisting of a father, mother and children, while three participants lived with extended family members, including grandparents, uncles, or aunts.

**TABLE 1:** Sociodemographics of participants.

Variable	*n*	%
**Age (years)**		
18–29	4	33
30–39	5	42
40–49	0	0
≥ 5	3	25
**Occupation**		
Unemployed	9	75
Employed	1	8
Pensioner	2	17
**Family structure**		
Nuclear	9	75
Extended	3	25
**Source of income**		
Social grant	9	75
Employment and social grant	1	8
Employment of spouse and/or partner	2	17
**Level of education**		
None	1	8
Primary school	4	33
High school	7	58
**Access to municipal utilities**		
Water in home	5	42
Electricity in the home	7	58

### Number of themes and sub-themes identified

Overall, 5 main themes and 22 sub-themes with 113 codes were identified, however, only 14 sub-themes were used for this article. The themes and sub-themes reported in this article are summarised in Table 2.

**TABLE 2:** Themes and sub-themes.

Themes	Sub-themes
Child feeding practices	Types of food fed to children
Concern for nutritional well-being of toddlers and children	Nutrition knowledge Primary caregiver’s feelings when they cannot feed their children properly
Social support for toddler and child feeding practices	Services at local clinics Support from partners and/or spouses Family
Financial restraints that impact toddler and young child feeding practices	Assistance from neighbours Reliance on social grants and wishing for employment Running out of funds for food Credit Money lenders
Access to household amenities	Water Electricity Vegetable gardens

### Child feeding practices

Foods that were given to the children who were no longer breastfed, included maize porridge (pap), milk, ‘juice’ (which on probing turned out to be cordials) and water. Vegetables and meat were only offered to the children once a week on Sundays. The most mentioned vegetables were cabbage, spinach, pumpkin, and carrots. Fruits were mostly fed to the children at the beginning of the month and considered treats, because the participants had money to purchase them at the time:

‘I give her usual food, pap and milk.’ (Participant 2)

‘I give the child water, I give the child pap and milk, and I give the child pap and meat.’ (Participant 3)

‘… when I have money I buy some fruits, and drinks and water.’ (Participant 12)

When the grandmother (Participant 10) had no meat, she fed the children maize porridge (pap) with oil previously used to prepare the meat (referring to drippings from the meat preparation):

‘Sometimes, if we don’t have any meat. I will just put a little fat, the fat that was left from the meat. Then I just put in the pap, and we eat it. Only when I get paid, I cook veggies.’ (Participant 10)

Other starchy foods mentioned were bread, cornflakes, Weet-bix™ (breakfast cereal), instant noodles, rice, and potatoes. The participants reported that they fed their children these foods because it would make the child grow, it was what they could afford, it was the food they were sure their child would eat, and they believed it was healthy. It was clear from their comments that the participants were aware of food groups and the importance of feeding their children healthy food, mostly because of the support they received within their community.

### Concern for the nutritional well-being of toddlers and children

Another theme that was identified was participants’ concern for the well-being of their children.

The participants seemed to prioritise the need to feed their children:

‘I can go bed hungry, but not the child.’ (Participant 1)

‘Yes, I have [*gone to bed without eating food*], and feel bad because I am a diabetes person. And sometimes I wake up very weak.’ (Participant 4)

‘[*S*]sometimes we really don’t have … I go borrow money from the people who lend money. Or I go ask people who I know … to buy little food [*kossies*].’ (Participant 7)

‘I try by all the means my child must not go to bed without any food.’ (Participant 11)

Furthermore, it was clear from participants’ comments that they had nutrition knowledge that the participants showed that they were aware of food groups and the importance of feeding their children healthy food:

‘Most of the time. I do not give the child gas cold drink. Because the gas is not good for the children. I give 100% cold drink, or I buy the cold drink that you mix with water and give them that to drink. And little sweets. And little of the chips …’ (Participant 1)

‘There are some that gives them allergies, even sweets give them allergies …’ (Participant 1)

‘… milk is nutritious, for child’s bones to be strong.’ (Participant 1)

‘What they can eat more of is meat. And with meat, I use chicken meat, and the white meat parts, not the skin and the fat. Because that is not good, even for adults it is not good.’ (Participant 1)

‘But the vegetables I think are 100%.’ (Participant 1)

‘Because water is very important for the body. And pap makes her grow [*she laughs*]. Most of the time, every time that I cook there must be cooking oil, it is the junk food. It is not the healthy food. Vegetables are the healthy food.’ (Participant 2)

‘The pap builds his body for weight gain and so on. The cereal is for energy and the iron that the child needs. Because the vegetables and fruit is very healthy for the up-growing of the child. The “opkoms” of the child. Some vegetables gives iron and vitamins and so on, and a lot of appetite. Then I don’t need to go the clinic for medicine and so on.’ (Participant 8)

‘Like I understand the vegetables are to build her “immune system,” so she can stay healthy. Most of the time the children, for example if I don’t give her then she just eats the sweets and chips then she will yawn, she her stomach runs, and she will become sick. So I realised if I can give her more fruit and vegetables then I can her body is alright … so if I don’t give vegetables, she loses weight and she doesn’t want to eat. Because if she gets more sweets, then she doesn’t want to eat her usual food.’ (Participant 9)

‘Because the Weet-bix, it has those the things, the calcium, the iron, it has zinc, they are affect the growing of the child.’ (Participant 11)

‘Okay, pap and milk is to give the children calcium, the bones must grow, he must grow stronger. And he likes too much pap and milk, and he don’t like meat. And he go to school and have that energy, and always carrots for the eyes, he must see clearly, yeah.’ (Participant 12)

As sources of their nutrition information, seven participants revealed that they had received nutrition advice from nurses at their local clinics. Seven participants also talked about receiving nutrition advice from family members, such as grandmothers, mothers, and neighbours (the older women in their communities), the matrons at the ECD centres, and television:

‘The clinic said I must not give, I must no more give the child thin porridge. The child must not eat light food. He must only eat the things that keep him strong.’ (Participant 4)

‘The clinic did say we must give him pumpkin, potatoes, cooked veg and thin porridge.’ (Participant 6)

‘In clinic they give some advice.’ (Participant 12)

‘Yeah, there is someone who tells me I must give my child pap… It is my grandmother.’ (Participant 2)

‘Because she is the oldest person and knows how to raise the child. That is why I listen to her.’ (Participant 3)

‘It’s the grandmother from next door. Because my mother has passed away.’ (Participant 4)

‘And here at the school. They say we must change the diet for the children. Today if they are eating this, then tomorrow the child must eat something else.’ (Participant 10)

‘There are sometimes the TV will discuss food, what the children must eat, and nutrition. I take the advice from the TV usually.’ (Participant 1)

‘I look at the TV. I watch the TV. How the people give their children food.’ (Participant 5)

However, the participants expressed some concerns related to the foods that they considered to be healthy for their children:

‘But the pap. Starch!’ (Participant 1)

‘Usually, the doctor says you must not eat pap every day. But under the circumstances we are living now, we must eat pap. We can’t afford the other food. So with my thinking, if you eat the pap with milk that starch will not be too much for the child.’ (Participant 1)

‘Even starch is not so healthy, but yeah. But the only things that is healthy is the water.’ (Participant 2)

‘Yes the pap and milk, but sometimes the meat is not healthy. Because I’m sometimes cooking with spices and the others have too much fat.’ (Participant 2)

‘Because pap and sugar is not healthy. At least pap and spinach. Yeah is healthy for the body, mind; everything for the body is good. And meat is not also good for the body. The sugar is going to make him sick at school, tired, they don’t have energy to listen to the teacher. At least when I give her pap and milk in the morning, yeah, she will be like other children in the class. She won’t get tired. Joh, it’s not healthy the meat. It’s too much fat. It’s not right for the body.’ (Participant 5)

The participants were concerned that having insufficient funds influenced the types of foods that they fed their children, and when they were able to offer vegetables and fruits to their children:

‘I must give him vegetables. But, I only give vegetables at month-end. If I don’t have, then we live like that until month-end. Potatoes, beetroot and apples. If I don’t have, then I just leave it.’ (Participant 3)

‘Sometimes I get in a week, three weeks we didn’t eat healthy food like spinach so on. Sometimes it’s a month, and we just eat starch. But when the money is enough, then we eat those vegetables and fruit now and then. I can say when it comes to the food, I make sure that there is, even when there is not really money. But when it comes to vegetables, I have a bit of a shortage. But bedtime, they go to sleep their tummies are always full.’ (Participant 8)

The amount of money that the participants had available also influenced the type of protein and how often protein was offered to their children:

Participant 8: ‘Sometimes it’s a month, and we just eat starch.’ (Participant 8)

Participant 2: ‘Just if I receive some little of the money, I buy Russians, you see …’ (Participant 2)

Participant 8: ‘For meat, for now mostly eat chicken because it is the cheapest. But so once in a month when the “uncle” had “piece-job,” we can have red meat, well actually white meat because it is pork and wors, is all I can say we eat. [*She giggles*] I can’t afford mutton, so I leave that.’ (Participant 8)

At the times when there was insufficient money for food, participants reported skipping meals so that their children could eat:

‘Nooo! No, I make plan for them to eat. If they eat, I don’t eat, I don’t have supper; about them, I have stress. I make plan for them to sleep with no empty stomach.’ (Participant 5)

‘Never, I try by all the means my child must not go to bed without any food.’ (Participant 11)

‘Even me I can sleep with the hunger, but only the child.’ (Participant 12)

However, not having sufficient funds for food was a very emotional experience for primary caregivers, and they described feelings of sadness, failure, and stress when they could not feed their children adequately:

‘I feel sad, too much.’ (Participant 2)

‘Heartbroken. Because I must go lend for my children?’ (Participant 3)

‘I don’t feel good, I feel like I’m failing my child.’ (Participant 4)

‘I feel so hard. It’s hard for me. Because sometimes they come from school and they say “we want pap.” I say, “joh we don’t have pap now, I will see later what I can do.” Sometimes they are crying, it’s not nice.’ (Participant 5)

‘Hurt, especially when I think my children must sleep with hunger.’ (Participant 7)

‘I feel sad [*heart sore*]. That I am not playing my role as a mother.’ (Participant 8)

‘I felt unhappy, because for myself, I don’t feel like my child can stay healthy. I am … I am not satisfied Ma’am, I am really not satisfied. Even though there is a bit, I am prepared I will give the child food. I can go sleep with hungry. Just as long as the child can sleep and eat.’ (Participant 9)

Participant 11: ‘I’m stressing, I’m feeling nervous because the child doesn’t get what always normally get at that time.’ (Participant 1)

‘It is stressful …’ (Participant 12)

One participant described how happy it made her when she was able to make a plan to give her child a fruit:

‘Because sometimes he sees another child with a banana then he want it too. And you do not have money at that time, how does the child feel. The child does not understand like me, like an adult when life is hard. But, I make sure that I must have R10 in the house so that the child can get fruit. Even if it is just once a week, I feel happy.’ (Participant 1)

### Social support for toddler and child feeding practices

This theme was related to the social support that participants accessed for feeding their young children. The first sub-theme was related to their use of and access to services at the clinics. They described that they received advice from the clinic staff on feeding to ensure that their children were healthy, growing and developing well:

‘I listened because it is a clinic that told me what food to feed the child when the child was six months old.’ (Participant 6)

However, their comments indicated that they did not always find the information practical and achievable within their circumstances:

‘Usually, the doctor says you must not eat pap every day. But under the circumstances we are living now, we must eat pap. We can’t afford the other food.’ (Participant 1)

‘Yeah, at the clinic they did … Sometimes I do it, sometimes not doing it … As I said, sometimes I do have those things that I must feed the child, sometimes I don’t have it.’ (Participant 2)

‘In the clinic, they give some advice, but ahh …’ (Participant 12)

Family, particularly grandmothers, were noted as a source of social support in feeding their young children:

‘Sometimes at my mother or mother-in-law. Yes, or borrow to add to the money.’ (Participant 8)

Neighbours (the community) also gave the mothers and primary caregivers social support in the form of advice regarding the feeding of young children:

‘Because she is the oldest person and knows how to raise the child. That is why I listen to her.’ (Participant 2)

‘She just told me that kids must eat pap and milk, must not give kids light food. And, I must give healthy foods, like spinach, fruits and veggies.’ (Participant 4)

‘Because she has grown many children.’ (Participant 4)

Finally, neighbours also supplied social support in the form of food assistance:

‘Yeah, like sometimes I don’t have mielie meal, so I just go to the next door.’ (Participant 4)

However, this kind of support was not without pitfalls, as a participant explained being scared that the neighbours might speak behind her back because they did not understand her circumstances:

‘Because like, other people they talk. Like “(*her name*), you’re getting two grants, but you don’t have …”; “They don’t know how we struggle as people.’ (Participant 12)

The third sub-theme is related to being able to depend on social support from relationship partners. Some participants talked about them not being able or willing to contribute financially to the household:

‘No, I have relations with the man. He does not do piece-job. I use the R100 to buy pap, I buy milk and I buy little meat to be satisfied when we eat.’ (Participant 3)

In this case, the boyfriend of the participant took a portion of the child grant money to buy things for himself:

‘Nothing [*he is not working*] … he’s just … No, we are just helping each other for the children.’ (Participant 12)

Two participants reported that their partners had some income and contributed to the household:

‘My husband has a job.’ (Participant 9)

‘My husband is working.’ (Participant 11)

In some cases, their partners could only contribute occasionally:

‘He is doing part-time jobs, there and there. Sometimes.’ (Participant 2)

‘No, the father … when the father was working, he cared for the child.’ (Participant 6)

‘Yes, he (boyfriend) works.’ … once in a month when the “uncle” had “piece- job …”’ (Participant 8)

The limited financial support from partners further contributed to the financial restraints of the household.

### Financial restraints that impact toddler and young child feeding practices

Financial restraints were identified as a theme, as it seemed that all the participants were affected, and it influenced their access to food for their children. As mentioned before, only two participants’ partners had jobs. The heavy reliance on social grants was therefore, identified as a sub-theme:

‘I’m living with the grant of the children …’ (Participant 12)

‘But now the father is without work. So we just live off the grant.’ (Participant 6)

All 12 participants reported that the source(s) of income they had access to needed to be more adequate for food. Five participants specifically mentioned that they would like to gain employment or a source of income to improve their food access:

‘What I can say the only thing I will need is the job. Then I can get the money and buy the healthy food you see.’ (Participant 2)

‘I don’t work. Sometimes I feel if my money, if I maybe only I had a job I could add to the grant money I get, support money that I get to just. Like most of the income.’ (Participant 8)

‘Ma’am, what I need is a job, an income. Because if I have an income, I can provide for the health of the child.’ (Participant 9)

‘So if I had a garden, I would have something. Most of the vegetables I would sell so that I could have an income.’ (Participant 8)

The low level of education among the participants was also identified as a sub-theme as it limits the types of jobs that the participants would be able to qualify for, and thus the level of income that they could generate should they find employment.

One participant wished for earning income outside of formal employment:

‘So, if I had a garden, I would have something. Most of the vegetables I would sell so that I could have an income.’ (Participant 8)

Running out of funds in the households for food and amenities affected all the participants at various levels. It also had an impact on the type of foods that they bought:

‘No, not often, it does not happen often. Just sometimes. Like I said, maybe towards month-end, you know things get finished.’ (Participant 1)

‘Like when it is 25 to 26 date of the month, we don’t have something to eat.’ (Participant 5)

‘[*She sighs*], probably so twice in a month.’ (Participant 7)

‘It happens during every time before the father is going to be paid. Maybe two weeks before.’ (Participant 11)

Not having sufficient income forced them to make other plans to access money for food and amenities. Two participants reported using store credit to purchase food and electricity when they had none:

‘I go to the shop and say, please help me with this and this, just for the child. I will pay you when I have money, then they help me … Then I go to the shop and make a loan so that the child can eat until I get money.’ (Participant 1)

‘I’m just going to the shops, and then they can give me credit. And then month-end I can pay.’ (Participant 11)

Nine participants also reported borrowing money to purchase food and electricity from people they know, or from lenders (so-called ‘loan sharks’):

‘No, I go borrow money from the people who lend money. Or I go ask people who I know.’ (Participant 7)

‘If my money is not there anymore, then I go to someone and borrow.’ (Participant 10)

‘From the loan lenders, the ones who borrow me. The money I don’t borrow for the food, because I used to have the garden …’ (Participant 12)

However, they also reported that the lenders charged high-interest rates:

‘Sometimes I do have, when I borrow the money for electricity, to whoever, I must give that money with the interest. So that is giving me a problem.’ (Participant 10)

‘Or sometimes I go to someone to lend me money so that I can pay that person … A loan shark … Yes, with the interest or double.’ (Participant 11)

‘And when I give it [*back*], I give it with the interest.’ (Participant 12)

Having to borrow and repay the money at the end of the month, often at high interest rates, further tapped into the little money the participants had available for food. Participants observed that borrowing money is something that they had to do to ensure that their children had food to eat and that their household had electricity to store and cook food:

‘Then I have to go and find some money from someone so that they can eat.’ (Participant 5)

‘I have to go loan some money to buy some food.’ (Participant 5)

‘Then I will see later where I can go and find some money for electricity. So that we don’t sleep in the dark.’ (Participant 5)

‘I lend money there. So I can buy electricity. Then I repay it the end of the month.’ (Participant 6)

### Access to household amenities

The household facilities (utilities and resources) available to the participants influenced the foods they fed their children. Only five participants had access to tap water in or outside the household. The other seven participants explained their challenges with access to water, including the frequent unavailability of municipal water even when they had the facilities for tap water and the frustrations and challenges of having to collect water elsewhere:

‘Yes, I do have water, but I don’t have water in the yard. I’m fetching from another place. I use four buckets.’ (Participant 4)

‘No, we don’t have water. We go down, we go to get water.’ (Participant 5)

‘Yes, there is water. Sometimes there isn’t water, then I must go far to the spring to fetch water.’ (Participant 6)

‘I um, collect water because we get water twice a day at home. So when the pumps open I fill the buckets and boil most of the water we drink, I boil it.’ (Participant 8)

Participant 11: ‘Sometimes we do have water, and sometimes the water is not there. So we and go and fetch water …’ (Participant 11)

‘The water is the problem of the farming, hence they told us the whole [*the name of the town*] is the problem with water. Sometimes, you know you have water, sometimes you don’t have water. It’s a problem of Kopanong.’ (Participant 12)

‘Sometimes the water is out, sometimes it’s not out and then the garden is dying.’ (Participant 12)

Collecting water increases the electricity needed to boil the water to ensure it is safe to drink. The participants explained their perception of the expense of electricity:

‘You buy R300 today, not even a week it is finished, because the electricity is little. Then you have to buy again … I think within a month, if I add up those receipts, then I bought R1000 electricity. Because the end of the month I leave some money to in between.’ (Participant 1)

The necessity for electricity was linked once again to having to borrow money:

‘Yes, and if I don’t have, I go ask for next door, and I’ll ask for money.’ (Participant 4)

‘No, when I don’t have electricity, I go and lend at someone, the backdoor. Then she lends to me.’ ‘I lend money there. So I can buy electricity. Then I repay it the end of the month.’ (Participant 6)

‘Sometimes, if I don’t have, I can go to the other side to borrow money to buy electricity.’ (Participant 9)

‘Sometimes I don’t have, but I make plan.’ ‘No, because when I don’t have money for electricity, I borrow the money.’ (Participant 10)

‘When the electricity is short or is not there, I go to borrow the money to buy electricity.’ (Participant 11)

‘I borrow the money maybe for electricity, then it’s finished.’ (Participant 12)

Participants reported cooking multiple meals on certain days in an attempt to save water and electricity:

‘I’m cooking more food to save electricity.’ (Participant 5)

Six participants explained that having a vegetable garden would improve their access to healthy foods. The lack of access to water hindered the participants from maintaining vegetable gardens:

Participant 12: ‘I used to have garden but it’s just because of water now, I’m struggling. Sometimes the water is out, sometimes it’s not out, and then the garden is dying.’ (Participant 12)

Regarding vegetable gardens, participants seemed to appreciate the value thereof to add diversity and healthy food to the household diet but reported that they lacked access to seeds:

‘Yes, I have, but now it’s no longer growing. I don’t have any more seeds. I was doing spinach there … I was cooking it for us, if we don’t have food. The other one I give my neighbours, because sometimes they help me. So when I have, I give them.’ (Participant 4)

‘I think I will need something like gardening so that they can eat healthy food.’ (Participant 5)

‘Um, most of my problem is vegetables. So if I had a garden I would have something. For the vegetable garden, I need plants, I need the seeds.’ (Participant 8)

‘But, even if can’t work, then a vegetable garden in the yard. Then I can at least cook the vegetables for the house.’ (Participant 9)

‘I need someone who can supply me with the seed, the planting so that I can plant in the garden. So that every time I need to cook and I can go there and just take things.’ (Participant 11)

‘Then I will just grow there, get spinach and cook it. And then my children just eat.’ (Participant 12)

As a household appliance or facility, televisions were reported as a source of information about feeding their children:

‘There are sometimes the TV will discuss food, what the children must eat, and nutrition. I am very interested by the TV, then I take advice from the TV. When I use the advice that I got from the TV, I see it works for me, I don’t know about the other people. But when I see it works for me, and I see other people with their children, then I tell them I read this and that from the TV, so do this and that and then they do it. Then it works.’ (Participant 11)

‘I watch the TV. How the people give their children food. I see they give them oats, in the morning, porridge, bread. Then in the afternoon they give them pap and milk. Then they will play, then they will give them some food to eat, milk to drink. Something like that. And vegetables.’ (Participant 5)

## Discussion

This exploratory qualitative study examined the lived experiences and beliefs of mothers and primary caregivers of children attending ECD centres and the factors that affect their feeding practices in the Xhariep district situated in the Free State province of South Africa.

The Xhariep district has a population of 121 687 people, of which 71.5% are black African, 18.2% are mixed race, and 9.4% are white, with other population groups making up the remaining 0.9% (Cooperative Governance & Traditional Affairs [COGTA] 2020:8).

The level of education of an individual often leads to a better household food security status because of the combined effect of better knowledge of nutrition and better access to food because of a higher income (De Cock et al. 2013:275). Food poverty is the inability to afford or access food to make up a healthy diet (UK Department of Health 2005: cited by Sonnino & Hanmer 2016). Only 6.4% of the adults in this municipality have some form of post-school education, 20.7% have completed school, 33.3% have some secondary education but did not complete school (COGTA 2020:21). The median level of education of the participants in this study was grade nine. Two of the participants who were grandmothers only had primary school level of education. This fact is evidence of the disparities caused by the apartheid era in South Africa, and the further lack of resource allocation in the rural areas to improve adult education. The food poverty experienced by the mothers and primary caregivers in this study may be related to their educational deficiencies that reflect inability to budget for food purchases, as well as a lack of skills to prepare and cook food, as also determined by Midgley (2013:301).

Moreover, food poverty is influenced by increased food prices, resulting in an increased share of household income spent on food. This causes households in the lowest income groups to purchase and consume less fruit and vegetables and more processed food products. In this study, the food poverty of the mothers and primary caregivers resulted from the financial, social, cultural and educational factors affecting their lives (Sonnino & Hanmer 2016). Overall, 41.9% of the population of the Xhariep district are living in poverty (COGTA 2020:9), with an income of R760 per month (as indicated by Statistics SA (October 2023) (equal to 38.68 USD per month) (Cowling 2023:1). High unemployment rates and reliance on pensions or child support grants were identified, while a large percentage reported running out of money to purchase food (Walsh & Van Rooyen 2015). It is thus not surprising that the participants of this study reported running out of funds to purchase food and thus had to use coping strategies such as skipping meals, borrowing money from money-lenders, and using store credit to mitigate the periods of food insecurity and to ensure that their children had food to eat. This in line with the results of STATS-SA (2016), which indicated that female-headed households (25%) with young children are more likely to reduce meal sizes than male-headed households (17.3%) (STATS-SA 2016:13).

Another study conducted in 2013 in Bophelong, a low-income peri-urban neighbourhood in the Vaal area of Southern Gauteng province indicated that as the Coping Strategy Index Scores/Household Food Insecurity Access Scale Scores of households increased, households relied statistically significantly more on so-called consumption soothing strategies (rationing and dietary change). These strategies included buying cheaper food, purchasing food on credit, limiting portions sizes at mealtimes, skipping meals, and adults consuming less to provide more food for smaller children (Grobler 2014:104).

The median age in the district is 26 years and the average household size is 3.0 persons per household, of which 37.6% are headed by women (COGTA 2020). Information gathered on intra-household food distribution during times of food shortages in the Limpopo province in 2011 found that female adults (18 years and older) would be the ones to eat less if there was a food scarcity, making them the most vulnerable category to food insecurity. Children under the age of five were the most likely to have enough food to eat, followed by children aged 5–18 years (De Cock et al. 2013:273). These findings are of concern as the mother is vulnerable to malnutrition in the form of hidden hunger and micronutrient deficiencies.

The employment opportunities are related to the policymaking and political domains of the 2020 UNICEF Determinants of Maternal and Child Nutrition framework for preventing malnutrition in all its forms. Policies should be focussed on creating an open, viable, and dynamic rural labour market with sustainable and equal opportunities for employment (De Cock et al. 2013:280) to further education and developing skills.

The findings of this study show how intertwined nutritious, diverse and safe foods in early childhood and a healthy environment, including access to basic health, water, hygiene, and sanitation services are. The lack of one of these affects the other in this community. The vicious cycle of lack of education, employment, and food clearly contribute to the hardships in the lives of the mothers, which, in turn, affect their children’s nutrition and health outcomes.

This study’s limitation lies in the inability to generalise finding to larger populations. Qualitative inquiries prioritise depth over breadth, with data saturation reached. The population’s homogeneity, sharing similar living, economic, and educational conditions, led to data saturation with a small participant count. Despite representing impoverished rural South African communities often overlooked in surveys, efforts were made to ensure transferability to similar populations. It is left to the reader to assess applicability to mothers in comparable rural settings in South Africa.

## Conclusion

This study revealed resource scarcity in the rural area, alerting clinic personnel to the dire state and minimal societal advancement in Xhariep district. It provides insights into the daily struggles of primary caregivers, including walking to fetch water, high electricity costs, financial restraints, and resorting to high-interest loan options to secure their children’s meals. Yet, the issue persists until decision-makers intervene, providing practical health education, access to basic water, and hygiene services, and enhancing employability skills in these rural areas.

According to this study, primary caregivers’ feeding practices were influenced by their level of education, employment status, and community support. The primary caregivers reported feeding the children mostly pap (maize porridge), milk and water. The diets were insufficient in vegetables and fruit, which places the children at risk of overweight, obesity and micronutrient deficiencies. Nutrition education interventions should aim to educate the older women in the community because the mothers rely on them for advice.

High electricity costs limited household funds, while water scarcity hindered vegetable gardening that could supplement purchased foods. Furthermore, social grants help purchase food and necessities for the child and household members, yet larger households faced heightened food insecurity. This study also reports on the high levels of unemployment of the adults in the household, leading to reliance on store credit and money lenders at interest, further straining the primary caregivers financially. Enhanced education and financial literacy among primary caregivers could mitigate these challenges, fostering long-term financial stability.

This study creates awareness of the lack of social progress and access to resources in Xhariep district’s rural communities, urging further investigation in similar impoverished regions. The results may contribute to the body of literature on maternal feeding practices, fostering improvements in social progress and access to basic health and sanitation services, enhancing both maternal feeding practices and child survival.
